# Thymoquinone-Loaded Electrospun Fibrous Mats as Advanced Wound Dressing Materials

**DOI:** 10.3390/pharmaceutics18060746

**Published:** 2026-06-17

**Authors:** Magdalena Paczkowska-Walendowska, Elwira Sieniawska, Zbigniew Krasiński, Judyta Cielecka-Piontek, Krystyna Skalicka-Woźniak

**Affiliations:** 1 Department of Pharmacognosy and Biomaterials, Poznan University of Medical Sciences, Rokietnicka 3, 60-806 Poznan, Poland; mpaczkowska@ump.edu.pl (M.P.-W.); jpiontek@ump.edu.pl (J.C.-P.); 2 Science-Bridge Sp. z o.o., Chociszewskiego 24/8, 60-258 Poznan, Poland; 3 Department of Natural Products Chemistry, Medical University of Lublin, ul. Chodźki 1, 20-093 Lublin, Poland; elwira.sieniawska@umlub.edu.pl; 4 Clinic of Vascular Surgery, Poznan University of Medical Science, Dluga 1/2, 61-848 Poznan, Poland; zkrasinski@ump.edu.pl

**Keywords:** electrospun nanofibers, wound healing, drug delivery, polyvinylpyrrolidone (PVP), polycaprolactone (PCL), hydroxypropyl-β-cyclodextrin (HPβCD), controlled release, mucoadhesion, antioxidant activity, anti-inflammatory activity

## Abstract

**Background:** Thymoquinone (TQ), a bioactive compound derived from *Nigella sativa* L., exhibits promising antioxidant, anti-inflammatory, and wound-healing properties; however, its clinical application is limited by poor solubility and instability. **Methods:** In this study, three electrospun nanofiber systems based on different polymeric matrices, PVP (N1), PVP/HPβCD (N2), and PVP/PCL (N3), were developed as potential wound dressing materials for controlled TQ delivery. **Results:** All formulations produced uniform nanofibrous structures with TQ molecularly dispersed within the polymer matrix, as confirmed by SEM, XRPD, and FTIR analyses. The composition of the nanofibers significantly influenced their physicochemical and functional properties. The N2 system, containing hydroxypropyl-β-cyclodextrin (HPβCD), exhibited the smallest fiber diameter (~208 nm), the fastest drug release, and enhanced antioxidant and anti-inflammatory activity due to improved TQ solubility. In contrast, the N3 system, incorporating polycaprolactone (PCL), formed thicker fibers (~1089 nm) and demonstrated sustained release behavior, the highest mucoadhesion, and the most pronounced wound-healing effect (90% closure after 24 h). Stability studies revealed that HPβCD significantly improved TQ resistance to thermal, humidity, and photolytic degradation, whereas the PVP-based system without stabilizers showed the lowest stability. Principal component analysis (PCA) confirmed that nanofiber performance is governed by two key factors: drug availability and sustained release combined with bioadhesion. Importantly, wound-healing efficiency correlated more strongly with the latter. **Conclusions:** The results demonstrate that rational design of polymer composition enables modulation of TQ delivery and biological response. Among the tested systems, PVP/PCL nanofibers appear to be the most promising candidates for wound-dressing applications due to their ability to provide sustained drug release and enhance tissue regeneration.

## 1. Introduction

Chronic and acute wounds represent a major clinical challenge due to their susceptibility to infection, prolonged inflammation, and impaired tissue regeneration [1]. An ideal wound dressing should provide a moist environment, protect against microbial contamination, and deliver therapeutic agents that support the different phases of the healing process [2]. The unique characteristics of electrospun nanofibers, including their extensive surface area, customizable pore structure, biomimetic morphology, and capability for sustained incorporation and release of active substances, make them attractive candidates for wound-healing applications [3,4,5,6,7].

Thymoquinone (TQ), the main bioactive constituent of *Nigella sativa* L. seed oil, exhibits a wide range of pharmacological activities, including antioxidant, anti-inflammatory, antimicrobial, and wound-healing properties [8,9]. These biological effects make TQ a promising candidate for topical wound therapy [10]. However, its clinical application is limited by poor aqueous solubility, sensitivity to light and oxygen, and chemical instability, which result in reduced bioavailability and rapid degradation [11]. Therefore, the development of suitable carrier systems capable of stabilizing TQ and enabling its controlled release is essential for its effective use in wound management.

Various nanocarrier-based approaches have been explored to improve the therapeutic performance of TQ, including lipid-based systems [12], polymeric nanoparticles [13], and hydrogels [14]. Recently, electrospun nanofibers have emerged as attractive platforms for the topical delivery of TQ. Several studies have reported the fabrication of TQ-loaded nanofibers from different polymers, mainly focusing on their antimicrobial or general wound-healing performance. For example, TQ has been incorporated into polymeric nanofibers, such as polycaprolactone (PCL)/chitosan [15] or polyvinyl alcohol-based systems [16], demonstrating enhanced antibacterial activity. These studies confirmed the feasibility of embedding TQ into fibrous matrices and highlighted the potential of nanofibers as local delivery systems for this compound.

Despite the growing interest in TQ-loaded nanofibers, most available studies focus primarily on selected aspects of biological performance, without a comprehensive evaluation of the interplay between formulation composition, physicochemical properties, drug release behavior, and stability. In particular, the combined impact of solubility-enhancing strategies (e.g., cyclodextrin complexation) and hydrophobic matrix modification on the functional performance of electrospun systems remains poorly understood. To the best of our knowledge, a systematic comparison integrating dissolution kinetics, mucoadhesion, stability under stress conditions, and biological activity of TQ-loaded nanofibers has not yet been reported.

Although several studies have reported TQ-loaded electrospun nanofibers, most investigations have focused on a single formulation approach and a limited number of performance parameters. To the best of our knowledge, a direct comparison of hydrophilic (PVP), cyclodextrin-containing (PVP/HPβCD), and sustained-release (PVP/PCL) electrospun systems loaded with TQ, combined with a comprehensive evaluation of dissolution, stability, bioadhesion, biological activity, and wound-healing performance, has not been reported previously. This work goes beyond previously published studies by providing a comprehensive evaluation of TQ-loaded nanofibers. In particular, the incorporation of hydroxypropyl-β-cyclodextrin (HPβCD) was investigated as a strategy to enhance TQ solubility and stability via inclusion complex formation [17], while the addition of PCL was used to modulate hydrophobicity, bioadhesion, and release [18].

The novelty of this study lies in the systematic comparison of these formulation strategies and the integration of physicochemical, biopharmaceutical, and functional data, further supported by multivariate analysis (PCA) to identify key factors governing system performance. This approach provides new insight into the relationship between material design and therapeutic efficacy, highlighting the critical role of controlled release and bioadhesion in wound-healing applications and offering a rational basis for the development of advanced nanofibrous dressings for poorly soluble bioactive compounds.

## 2. Materials and Methods

### 2.1. Chemicals and Reagents

Thymoquinone (≥98%), polyvinylpyrrolidone K30, hydroxypropyl-β-cyclodextrin (average Mw ~1460), and polycaprolactone were supplied from Sigma-Aldrich (Poznan, Poland). Reagents for activity assays (2,2-Diphenyl-1-picrylhydrazyl (DPPH), sodium chloride, bovine serum (BSA), hexadecyltrimethylammonium bromide (CTAB), hyaluronic acid (HA), and hyaluronidase enzyme) and for dissolution studies (phosphate buffer) were obtained from Sigma-Aldrich (Poznan, Poland). High-quality pure water and ultra-high-quality pure water were prepared using a Direct-Q 3 UV Merck Millipore purification system (Merck Millipore, Darmstadt, Germany).

### 2.2. Preparations of Nanofibers

Electrospinning was conducted using an NS + NanoSpinner Plus system (Inovenso Ltd., Istanbul, Turkey). The polymer solutions described in Table 1 were placed in 5 mL syringes equipped with stainless steel needles with an internal diameter of 0.8 mm. The process was performed under an applied voltage of 27 kV, a solution flow rate of 2 mL/h, and a needle-to-collector distance of 12 cm. Electrospinning was performed at 25 ± 2 °C and 40 ± 5% relative humidity. Environmental conditions were monitored throughout the process to ensure reproducibility of nanofiber production. The resulting nanofibers were deposited onto aluminium foil and subsequently dried for 24 h to eliminate any remaining solvent.

### 2.3. Scanning Electron Microscopy (SEM)

The morphology of the nanofiber surfaces was analyzed by scanning electron microscopy (SEM) with a Quanta 250 FEG microscope (Thermo Fisher Scientific, Waltham, MA, USA). Before observation, the specimens were coated with a thin gold–palladium layer to improve electrical conductivity. Fiber diameters were measured from the SEM images using ImageJ software (version 1.54).

### 2.4. X-Ray Powder Diffraction (XRPD)

X-ray diffraction patterns were recorded using a Bruker D2 Phaser diffractometer (Bruker, Berlin, Germany) equipped with CuKα radiation (λ = 1.54060 Å), operating at 30 kV and 10 mA. Data were collected over a 2θ range of 5–40° with a step size of 0.02° and a counting time of 2 s per step.

### 2.5. Fourier Transform Infrared Spectroscopy (FTIR)

FTIR spectra were acquired in absorbance mode using an IRTracer-100 spectrophotometer (Shimadzu, Kyoto, Japan) with LabSolutions IR software (v.1.86 SP2). The analyses were performed over the spectral range of 400–4000 cm^−1^ at a resolution of 4 cm^−1^, applying Happ–Genzel apodization and averaging 400 scans.

### 2.6. Drug Content and Loading Efficiency

The thymoquinone (TQ) content in the electrospun nanofibers was determined using the validated HPLC method. The Kinetex-C18 column (100 × 2.1 mm, 5.0 μm) was used as a stationary phase (Phenomenex, Warsaw, Poland), while a mobile phase was composed of 0.1% formic acid and acetonitrile (80:20 *v*/*v*) with a flow rate of 1.0 mL/min. A diode-array detector was set to a wavelength maximum (λ_max_) of 254 nm. The column was set at 30 °C. Accurately weighed samples of nanofibers were dissolved in methanol, filtered, and analyzed by HPLC.

Drug loading efficiency (LE) was calculated as the ratio of the experimentally determined TQ content to the theoretical TQ content based on the formulation composition according to the following equation:

LE (%) = (Measured TQ content/Theoretical TQ content) × 100

All measurements were performed in triplicate, and results were expressed as mean ± SD.

### 2.7. Dissolution Behavior

Dissolution tests of the electrospun nanofibers were performed using an Agilent 708-DS dissolution apparatus (Agilent, Santa Clara, CA, USA) equipped with a standard basket system, operated at 37 ± 0.5 °C and 50 rpm. The nanofibers were immersed in 30 mL of phosphate buffer at pH 5.5 to simulate the slightly acidic conditions of normal human skin (pH 4.7–5.6) and the wound microenvironment, which shifts toward mild acidity during the early inflammatory and proliferative stages of the healing process. At specified time points, aliquots of the dissolution medium were withdrawn and filtered through a 0.45 µm nylon membrane. Each experiment was performed in triplicate. TQ concentrations were determined by HPLC.

The dissolution data were fitted to zero-order, first-order, Higuchi, and Korsmeyer–Peppas models according to the following equations:

zeroorderequation:F=k×t

first-orderequation:lnF=k×t

$$\mathrm{Higuchi}\mathrm{equation}:F=kt^{1/2}$$

$$\mathrm{Korsmeyer}\text{-}\mathrm{Peppas}\mathrm{equation}:F=kt^{n}$$

where *F* is the fraction of released drug, *k* is the constant connected with release, and *t* is the time.

### 2.8. Mucoadhesive Properties

A viscometric method was used to quantify the strength of the bioadhesive bond between mucin and polymeric material, as previously described [19].

### 2.9. Determination of Nanofibers Biological Activities

#### 2.9.1. Antioxidant Activity

Antioxidant activity was assessed using the DPPH (2,2-diphenyl-1-picrylhydrazyl) radical scavenging assay according to a previously described procedure [20]. Briefly, 25 μL of each sample was mixed with 175 μL of a methanolic DPPH solution (3.9 mg/50 mL methanol). The mixtures were vortexed and incubated in the dark at room temperature for 30 min. Absorbance was measured at 517 nm. Control samples consisted of 25 μL of water mixed with 175 μL of methanol, while the blank contained 25 μL of water and 175 μL of DPPH solution. All experiments were carried out in nine replicates. Ascorbic acid was used as a positive control.

#### 2.9.2. Anti-Inflammatory Activity

Hyaluronidase inhibitory activity was evaluated using a turbidimetric method described previously [20]. Briefly, the reaction mixture consisted of hyaluronidase solution (30 U/mL), acetate buffer (50 mM, pH 7.0, containing 77 mM NaCl and 1 mg/mL albumin), acetate buffer (pH 4.5), and the tested sample. Following incubation at 37 °C for 10 min, hyaluronic acid solution (0.3 mg/mL) was added, and the mixture was further incubated at 37 °C for 45 min. The remaining undegraded hyaluronic acid was precipitated with 2.5% CTAB in 2% NaOH and incubated at room temperature for 10 min. Turbidity was measured at 600 nm. All experiments were performed in six replicates, and β-escin was used as a positive control.

### 2.10. Cytotoxicity Assay and Wound-Healing Properties

Cytocompatibility of the tested formulations was evaluated using the MTT assay on human skin fibroblasts (Hs27). Cells were seeded into 96-well plates at a density of 1 × 10^5^ cells/mL and incubated for 24 h. Subsequently, the culture medium was replaced with medium containing the tested samples, and cells were incubated for an additional 24 h. Control cells received sample-free medium. After incubation, cell viability was determined using the MTT assay based on the reduction of MTT to formazan crystals by metabolically active cells. Absorbance was measured at 570 nm using a microplate reader. All experiments were performed in triplicate and repeated independently twice [21].

Hs27 cell line was purchased from the ATCC (Manassas, VA, USA) and maintained in DMEM– high glucose medium supplemented with 10% FBS, penicillin (100 U/mL), and streptomycin (100 µg/mL). The cells were cultured in a humidified atmosphere at 5% CO_2_ and 37 °C. On the day of the experiment, cells were collected from monolayers with trypsin/EDTA and seeded onto a 6-well plate at a concentration of 1 × 10^5^ cells/mL. When the cell confluency reached ~90%, a vertical linear scratch was performed with a sterile pipette tip. Cells were washed with PBS, and fresh medium (control group) or medium containing N1–N3 formulations at a thymoquinone-equivalent concentration of 10 µg/mL was added to the respective wells. The concentration of 10 µg/mL was selected based on preliminary experiments and literature data indicating biological activity of thymoquinone while maintaining acceptable fibroblast viability. Images of the scratch were taken at 0 h and 24 h using an Olympus CKX53 microscope coupled with an XM10 digital camera (Olympus, Warsaw, Poland). The scratch area at the beginning of the experiment (0 h) was considered 100%. The open wound area was measured using ImageJ version 1.54p (NIH, Bethesda, MD, USA). Wound closure (in %) was calculated using the following formula:

$$Closed\;wound\;area\;\left(\%\right)=\;\frac{open\;wound\;area\;at\;0\;h-open\;wound\;area\;at\;24\;h}{open\;wound\;area\;at\;0\;h}\;\times \;100\%$$

Results were expressed as the mean percentage of wound closure ± SD.

### 2.11. Stability Tests

Stability studies of the obtained nanofibers were conducted using three experimental models:

- Thermal stress testing: Samples were stored at 60 ± 2 °C for 14 days under constant relative humidity.
- Accelerated degradation under high humidity conditions: Samples were kept for 7 days in a climatic chamber at 25 ± 2 °C and 75 ± 5% relative humidity (RH).
- Photostability assessment: Samples were exposed to UV/Vis radiation in a photochemical chamber, with UV light at 365 nm and visible light at 420 nm for 2.5 h.

In all the above conditions, the TQ content was determined by HPLC using the previously developed and validated method, after prior dissolution of the nanofibers in an appropriate solvent (methanol). The results were expressed as a percentage of the initial TQ content.

### 2.12. Statistical Analysis

Statistical analyses were conducted using Statistica software (version 13.3). Differences between groups were assessed by one-way analysis of variance (ANOVA), followed by Duncan’s and Tukey’s post hoc tests for multiple comparisons. Statistical significance was established at *p* < 0.05. Correlation analysis and principal component analysis (PCA) were performed using PQStat software (version 1.8.4.142, 2022).

## 3. Results and Discussion

**Figure 1:**
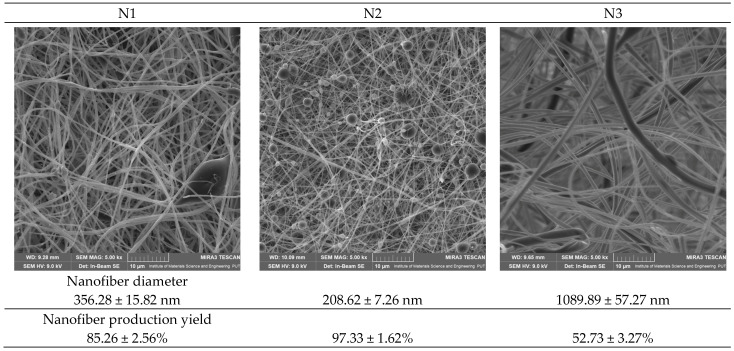
SEM images of nanofibers N1-N3 and their diameters. Scale bars = 10 μm.

Optimizing the electrospinning process for three nanofiber systems containing thymoquinone (TQ) enabled the fabrication of stable fibrous structures with distinct morphological characteristics and production yields. SEM analysis confirmed the proper nanofiber structure in all systems and allowed the assessment of their uniformity, diameters, and the absence of potential defects (Figure 1).

Among the investigated formulations, the N2 system produced the thinnest and most uniform nanofibers, with an average diameter of approximately 208 nm and the highest production yield (97.33%). The presence of HPβCD in its composition likely enhanced the stability of the spinning solution, thereby facilitating nanofiber formation. The N1 system also exhibited good fiber quality, with an average diameter of approximately 356 nm and a high process yield (85.26%). In contrast, the largest fiber diameters were observed for the N3 system (approximately 1089 nm), placing this formulation at the boundary between the nano- and microscale fiber range, indicating that the presence of PCL increased viscosity and altered the rheological properties of the mixture, thereby reducing fiber formation efficiency (yield of 52.73%).

The obtained results confirm the effectiveness of the applied electrospinning parameters and demonstrate the capability of this technique to produce nanofibers incorporating a natural active compound, thymoquinone. The differences observed between the systems highlight the significant influence of polymer composition on nanofiber morphology and production efficiency, which may also affect the physicochemical and biological properties of the resulting nanofibers.

**Figure 2:**
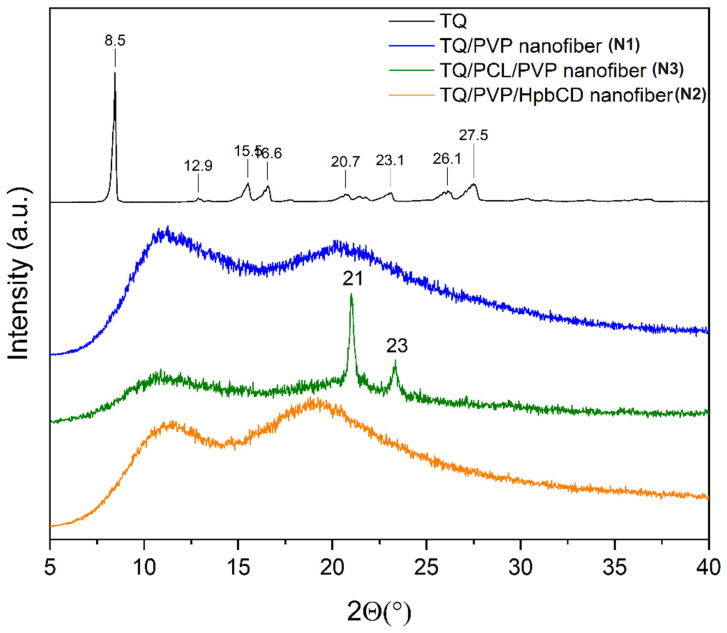
XRPD diffractograms of TQ and nanofibers N1–N3.

In the TQ diffractogram over 8–28°, Bragg peaks are observed, confirming its crystalline form (Figure 2) [22]. The absence of characteristic diffraction peaks of crystalline TQ in the XRPD patterns of N1, N2, and N3 indicates that TQ is present predominantly in an amorphous state within the polymer matrices. These findings suggest effective incorporation of TQ into the nanofibers and loss of its crystalline structure during the electrospinning process. The two peaks detected at 21° and 23° (for N3 nanofibers) are characteristic of PCL [23].

**Figure 3:**
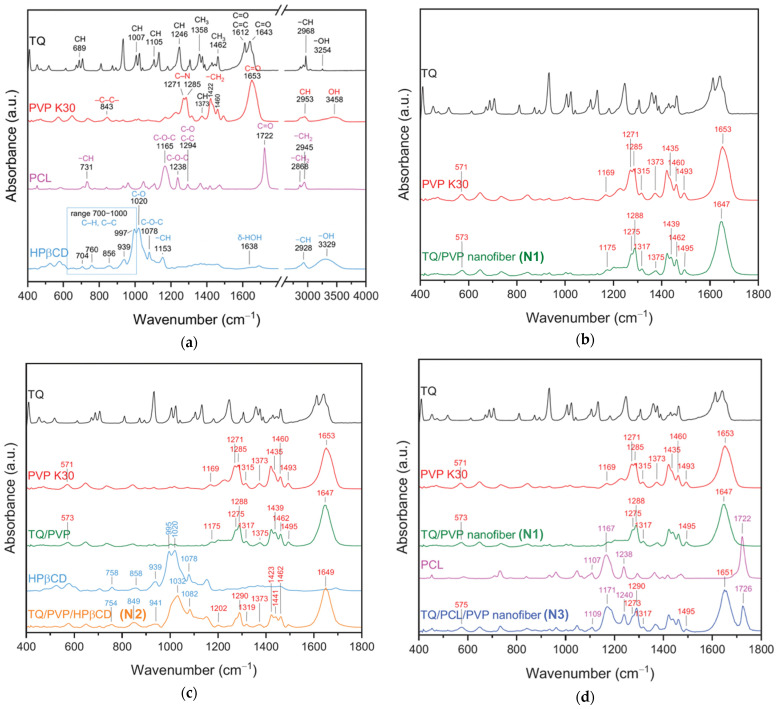
FT-IR analysis: TQ, PVP K30, PCL, HPβCD (**a**), N1 nanofiber (TQ/PVP) (**b**), N2 nanofiber (TQ/PVP/HPβCD) (**c**), and N3 nanofiber (TQ/PCL/PVP) (**d**).

The FT-IR spectrum of TQ (Figure 3a, black line) shows several characteristic absorption bands. A series of bands corresponding to C–H vibrations appear at 689, 1007, 1105, 1246, 1358, and 1462 cm^−1^. Two strong carbonyl stretching bands (C=O) are observed at 1612 and 1643 cm^−1^, confirming the presence of quinone groups. Additionally, a peak at 2968 cm^−1^ corresponds to aliphatic –CH stretching, and a broad band at 3254 cm^−1^ is attributed to –OH stretching, indicating minor hydroxyl-related interactions [24,25,26]. The PVP K30 spectrum (Figure 3a, red line) displays a characteristic C–C stretching band at 843 cm^−1^. Pronounced pyrrolidone-ring vibrations appear at 1271 and 1285 cm^−1^, assigned to C–N stretching and adjacent –CH_2_ bending (1373, 1460 cm^−1^). A strong C=O stretching peak from the lactam group is visible at 1653 cm^−1^. The polymer backbone shows CH_2_ stretching at 2953 and 2945 cm^−1^, and a broad –OH stretching band at 3458 cm^−1^, indicating the presence of hydrogen-bonding interactions [27]. PCL (Figure 3a, pink line) exhibits several characteristic peaks that appear between 1020 and 1294 cm^−1^, including C–O, C–O–C, and C–C stretching vibrations (1020, 1165, 1238, 1294 cm^−1^). A strong and sharp carbonyl (C=O) stretching peak is found at 1722 cm^−1^, which is the key marker of PCL. Methylene groups show stretching bands at 2945 and 2868 cm^−1^ [28,29]. The HPβCD spectrum (Figure 3a, blue line) displays typical saccharide-related absorptions. In the region 700–1000 cm^−1^, several peaks associated with C–H and C–C stretching are present, including bands at 704, 760, 856, and 939 cm^−1^, with a notable peak at 997 cm^−1^. Stretching vibrations of C–O and C–O–C groups occur at 1078 and 1153 cm^−1^, respectively. A band at 1638 cm^−1^ corresponds to δ-OH bending of bound water. The CH stretching band appears at 2928 cm^−1^, while the broad –OH stretching absorption associated with hydrogen bonding is located at 3329 cm^−1^ [4,27].

The FT-IR spectrum of the N1 nanofiber (TQ/PVP) (Figure 3b, green line) demonstrates characteristic bands originating from PVP K30. Noticeable peak shifts indicate intermolecular interactions between TQ and PVP. The absence or masking of characteristic TQ bands may be attributed to the amorphization of TQ and overlap with the PVP 30 polymer absorption bands. A low-frequency band at 573 cm^−1^ corresponds to skeletal vibrations of PVP. In the fingerprint region, several peaks characteristic of PVP (1169, 1271–1285, 1315 cm^−1^) shift to 1175, 1275, 1288, and 1317 cm^−1^, suggesting changes in the local chemical environment after incorporation of TQ. Additional bands at 1375, 1439, 1462, and 1495 cm^−1^ correspond to CH and C–C bending vibrations and show minor deviations from the pure PVP spectrum, indicating partial interaction with TQ molecules. The strongest marker of interaction is visible in the carbonyl region. The prominent PVP lactam C=O band, originally at 1653 cm^−1^, shifts to 1647 cm^−1^ in the TQ/PVP sample. This downward shift (red shift) indicates hydrogen bonding (interactions between the carbonyl group of PVP and functional groups of TQ).

The FT-IR spectrum of the N2 nanofiber (TQ/PVP/HPβCD) (Figure 3c, orange line) confirms the presence of PVP and HPβCD absence of the band of TQ. It confirms strong intermolecular interactions between the components. In the low-wavenumber region, bands at 754, 849, and 941 cm^−1^ can be ascribed to skeletal vibrations of the cyclodextrin ring and correspond to the characteristic signals of HPβCD at 758, 858, and 939 cm^−1^, whereas the slight shifts and changes in band shape indicate modification of the hydrogen-bond network upon complexation. The saccharide region between 900 and 1200 cm^−1^ shows a series of C–O and C–O–C stretching vibrations of HPβCD, with bands at 941, 995, ~1032, and 1082 cm^−1^ closely related to those of the pure carrier but partially broadened and overlapped with PVP contributions, which is consistent with the preservation of the cyclodextrin framework in the presence of the polymer. PVP-related bands located at range 1271–1285, 1315, 1373, 1435, 1460, and 1493 cm^−1^ in the spectrum of neat PVP are shifted in N2 nanofiber to 1290, 1319, 1373, 1441 and 1462 cm^−1^, similarly to but more pronounced than in the N1 nanofiber, reflecting changes in the environment of the lactam ring and CH groups due to additional interactions with HPβCD and TQ. In the carbonyl region, the strong C=O stretching vibration of the PVP lactam group, observed at 1653 cm^−1^ in the pure polymer and at 1647 cm^−1^ in the N1 nanofiber, appears as a single band at 1649 cm^−1^ in the N2 nanofiber. The persistence of this shifted and slightly broadened band, together with the disappearance of distinct crystalline TQ peaks, indicates the establishment of hydrogen bonding between the PVP carbonyls and hydroxyl groups of HPβCD as well as with the groups of TQ, and suggests that TQ is molecularly dispersed and at least partly included in the HPβCD cavity and/or strongly associated with the PVP matrix.

A weak band at 575 cm^−1^ is analogous to the low-wavenumber bands of neat PVP and confirms the polymeric matrix. In the fingerprint region, the bands at 1109, 1171, 1240, 1273, 1290, and 1317 cm^−1^ arise from overlapping C–O, C–O–C, C–N, and CH_2_ vibrations of PVP and PCL (Figure 3d). Their slight shifts compared with pure PVP (1169, 1271, 1285, 1315 cm^−1^) and pure PCL (1107, 1167, 1238 cm^−1^) suggest specific interactions, most likely hydrogen bonding, between the polymers and TQ. The band at 1495 cm^−1^ corresponds to combined C–C/CH_2_ bending modes from the PVP and is also slightly shifted relative to the individual components (PVP 1493 cm^−1^, TQ/PVP 1495 cm^−1^). The carbonyl region is particularly diagnostic: the strong PCL ester C=O stretching appears at 1726 cm^−1^, shifted to a higher wavenumber compared to neat PCL (1722 cm^−1^), while the PVP/TQ-related carbonyl band is seen at 1651 cm^−1^, shifted from 1653 to 1647 cm^−1^ in the single-polymer systems. These shifts, together with the broadening of the carbonyl envelope, indicate the formation of new hydrogen-bonded environments and confirm that TQ is molecularly dispersed and interacts with both PVP and PCL within the ternary formulation.

The experimentally determined TQ content was in good agreement with the theoretical composition of all formulations. Loading efficiency values exceeded 98%, indicating efficient incorporation of TQ into the nanofibrous matrices and negligible drug loss during the electrospinning process. These results confirm that the applied electrospinning conditions enabled effective encapsulation of TQ and ensured satisfactory content uniformity among the prepared formulations.

**Figure 4:**
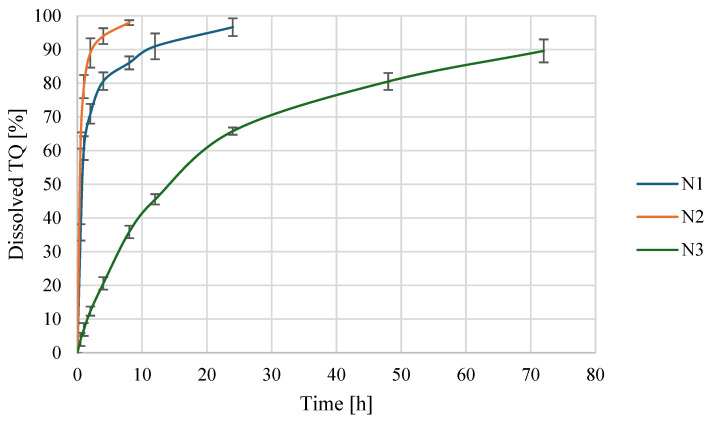
Dissolution profiles of TQ from nanofibers N1–N3.

Figure 4 presents the dissolution profiles of TQ released from nanofibers N1-N3 under simulated wound environment conditions. All formulations exhibited a rapid initial release phase followed by a more gradual release over time. The N2 system showed the fastest and most extensive release of TQ, attributed to the presence of HPβCD, which enhanced TQ solubility and promoted diffusion from the polymeric matrix [30]. The N1 formulation demonstrated a moderately fast release profile, consistent with diffusion-controlled transport from the PVP-based fibrous structure. In contrast, the N3 system exhibited the slowest and most sustained release behavior, reflecting the more hydrophobic nature of the PCL-containing matrix and its higher structural density [31], which hindered TQ diffusion.

To further elucidate the release mechanism, the experimental data were fitted to various kinetic models (Table 2). The dissolution data were analyzed using several commonly applied kinetic models. Although some models showed relatively higher R^2^ values than others, the overall fitting quality was moderate for N1 and N2 formulations. Therefore, the kinetic analysis should be regarded as indicative of general release trends rather than conclusive evidence of a specific release mechanism. The results suggest that diffusion-related processes contribute substantially to TQ release, while additional factors associated with the polymer matrix may also influence the release behavior [32]. In contrast, the N3 system exhibited the most controlled release profile and was best described by the Korsmeyer–Peppas model. The release exponent (n = 0.48) indicates anomalous (non-Fickian) transport, suggesting that TQ release is governed by a combination of diffusion through the fiber matrix and polymer relaxation-related processes [33]. This behavior is consistent with the presence of PCL, which contributes to a more complex and sustained release mechanism compared with the predominantly diffusion-controlled release observed for N1 and N2.

**Figure 5:**
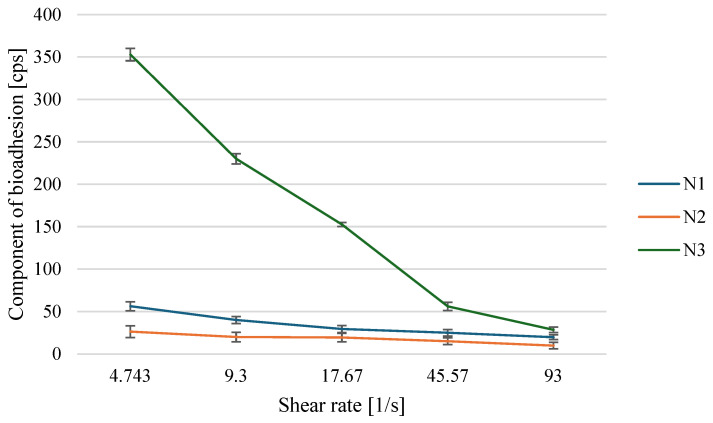
Component of bioadhesion of nanofibers N1–N3.

To assess the interaction between the nanofiber formulations and mucin, the bioadhesion component was measured over a range of shear rates. Figure 5 illustrates the dependence of bioadhesion on shear rate for systems N1–N3.

The N3 system exhibits by far the highest bioadhesion component, particularly at low shear rates (above 350 cps), which gradually decreases as the shear rate increases. The presence of PCL, a more hydrophobic polymer, promotes the formation of a denser, more structured matrix that can interact more strongly with mucin via both hydrophobic interactions and van der Waals forces. At the same time, this system exhibits higher viscosity and pronounced shear-thinning behavior, which accounts for its superior bioadhesive potential [34].

The N1 system shows intermediate values of the bioadhesion component compared with N2 and N3. PVP, a highly hydrophilic polymer, can form hydrogen bonds with mucin, resulting in moderate bioadhesion. However, the absence of additional structure-forming polymers results in a less crosslinked matrix and weaker interactions with mucin than those observed for the N3 system.

The N2 system displays the lowest bioadhesion component over the entire range of shear rates. The addition of HPβCD, although beneficial for the stabilization and solubilization of TQ, does not enhance bioadhesion, as cyclodextrins mainly act as carriers for lipophilic compounds rather than as bioadhesive polymers. Moreover, the formation of inclusion complexes with TQ may further reduce the availability of PVP’s functional groups and alter the system’s rheological properties, thereby weakening interactions with mucin [35].

The antioxidant activity of the tested sample was evaluated using the DPPH radical scavenging assay (Table 3). In the case of thymoquinone, the radical scavenging activity is associated with its quinone moiety, which can participate in redox cycling and form semiquinone intermediates. However, compared with polyphenolic antioxidants, thymoquinone is a relatively weak hydrogen donor, which explains its moderate effectiveness in the DPPH assay [36]. Literature data report IC_50_ values for pure thymoquinone in the range of approximately 70–150 µg/mL, whereas significantly higher IC_50_ values (in the mg/mL range) have been reported for extracts of *N. sativa* [37,38,39]. This indicates that pure thymoquinone exhibits stronger DPPH radical scavenging activity than complex extracts under comparable conditions. Incorporation of TQ into polymeric matrices resulted in changes in IC_50_ values, which can be attributed to dilution of the active compound and to limitations in its accessibility within the nanofibrous structure. The N1 system exhibited an IC_50_ value lower than that theoretically predicted based on the mass fraction of TQ, which may be ascribed to the good solubility and homogeneous dispersion of thymoquinone in the hydrophilic PVP matrix. In the presence of HPβCD, a decrease in IC_50_ relative to the theoretical value was observed, indicating a beneficial effect of cyclodextrin on the effective availability of TQ through the formation of inclusion complexes and enhancement of its solubility in the reaction medium. In contrast, the IC_50_ value of the N3 formulation was higher than expected, which may be attributed to the presence of hydrophobic PCL, which restricts the release and diffusion of thymoquinone into the solution and thereby reduces its effective participation in the reaction with the DPPH radical.

The anti-inflammatory activity of the developed nanofibers was evaluated by their ability to inhibit hyaluronidase (Table 3), an enzyme that degrades hyaluronic acid and is a key mediator of inflammatory processes in the extracellular matrix. TQ exhibited moderate anti-inflammatory activity, which is consistent with its well-documented pharmacological profile. Numerous studies have shown that TQ modulates inflammatory pathways by inhibiting pro-inflammatory mediators, cytokines, and signaling pathways such as NF-κB, as well as through antioxidant mechanisms [9]. Incorporation of TQ into nanofiber systems significantly influenced its apparent activity. The N1 formulation (TQ/PVP) showed reduced inhibitory potential, which can be attributed to the limited availability of TQ despite its molecular dispersion in the hydrophilic matrix. This effect is commonly observed for poorly soluble compounds embedded in polymer carriers, where diffusion into the biological environment becomes a limiting factor [40]. A clear improvement in anti-inflammatory activity was observed for the N2 system (TQ/PVP/HPβCD). This enhancement is attributed to the presence of hydroxypropyl-β-cyclodextrin (HPβCD), which forms inclusion complexes with hydrophobic molecules such as TQ, thereby significantly increasing their aqueous solubility and effective bioavailability. Cyclodextrins are widely used drug delivery excipients due to their ability to encapsulate lipophilic compounds within their hydrophobic cavity while maintaining overall water compatibility [41]. The N3 formulation (TQ/PVP/PCL) exhibited intermediate anti-inflammatory activity. The presence of polycaprolactone (PCL), a hydrophobic, semicrystalline polymer, likely limits the initial release of TQ, thereby reducing its short-term bioavailability. However, such systems are known to provide sustained drug release, which may support prolonged therapeutic effects rather than immediate activity [42].

The results confirm that the biological activity of the nanofibers is primarily governed by the availability of thymoquinone, whereas the composition of the polymeric matrix significantly modulates its apparent efficiency by affecting the solubility, dispersion, and release kinetics of the active compound [16].

The assay was performed as a preliminary screening step to evaluate the effect of the formulations on fibroblast viability and to select a non-cytotoxic concentration for subsequent wound-healing studies. Based on these results, a concentration of 10 µg/mL was selected for the scratch assay, as no adverse effects on cell viability were observed at this level; cell viability was >98%.

**Figure 6:**
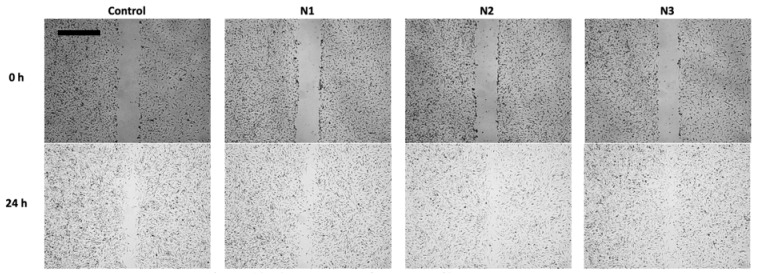
Representative images of wound-healing properties of N1-N3 nanofibers. Scale bars represent 500 μm.

**Figure 7:**
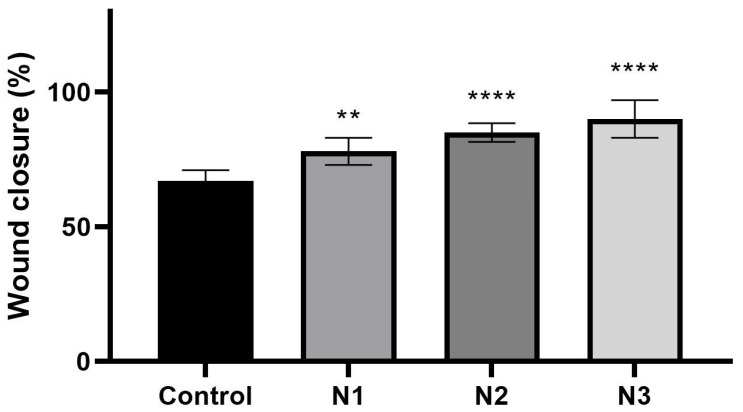
Wound-healing properties of N1-N3 nanofibers were observed after 24 h. The samples were tested at a concentration of 10 µg/mL. Results were statistically analyzed by ANOVA with a post hoc Tukey’s test. Statistical significance was designated as ** when *p* < 0.005 and **** when *p* < 0.0001 (vs. Control).

Wound-healing properties of the developed nanofibers were evaluated using a scratch assay on Hs27 fibroblasts. Representative microscopic images (Figure 6) showed a clear reduction of the scratch area after 24 h in all samples treated with TQ-loaded nanofibers compared to the control, indicating enhanced cell migration and wound closure. Quantitative analysis of wound closure after 24 h (Figure 7) demonstrated that all TQ-loaded nanofiber systems significantly enhanced fibroblast migration compared to the control. The control group reached 70% wound closure, whereas all treated samples exceeded this level. The N1 formulation moderately increased wound closure (78%; *p* < 0.005), indicating a beneficial effect of TQ delivered from the PVP matrix. A further improvement was observed for the N2 system (85% wound closure), with high statistical significance (*p* < 0.0001 vs. control). This confirms that incorporating HPβCD enhances TQ availability and promotes cell migration. The highest wound closure was achieved for the N3 formulation (90%), also with very high statistical significance (*p* < 0.0001 vs. control). This suggests that, despite its slower release profile, the presence of PCL provides more favorable conditions for sustained stimulation of cell migration, potentially translating into improved wound-healing performance. The results indicate that all nanofiber systems support wound healing, with the effectiveness increasing in the order: N1 < N2 < N3. This trend highlights the importance of controlled release and matrix composition in modulating the biological response.

In this study, a concentration of 10 µg/mL was tested. This concentration enabled a direct comparison of the different nanofiber formulations under standardized conditions. Nevertheless, the authors acknowledge that the wound-healing response may be concentration-dependent. Therefore, future studies should investigate a wider concentration range to establish dose–response relationships and determine the optimal therapeutic concentration for wound-healing applications.

To assess the stability of thymoquinone in the developed nanofiber systems, stress tests were performed under thermal, humidity, and photolytic conditions. The corresponding organoleptic changes and residual TQ content are presented in Table 4. The N1 system (PVP + TQ) exhibits the lowest stability among the investigated formulations. This is directly related to PVP’s properties: it is highly hygroscopic and readily absorbs water. The presence of moisture can catalyze hydrolytic reactions and accelerate TQ degradation. Moreover, in this system, TQ is not “physically protected” in any way, as PVP does not form stable complexes with it; rather, it acts only as a carrier. As a result, TQ molecules remain more accessible to light and moisture, leading to faster decomposition and loss of the active form. Photodegradation of TQ in the presence of PVP proceeds intensively, as evidenced by color changes (yellowing). The lack of a stabilizing component makes the N1 system the most susceptible to degradation.

The N2 system (PVP/HPβCD + TQ) demonstrates the highest quantitative stability of TQ across all tested conditions, including elevated temperature, increased humidity, and UV/Vis light. HPβCD plays a key role, forming an inclusion complex with TQ. Its cyclic, hydrophobic cavity can encapsulate the TQ molecule in a capsule-like structure, thereby limiting its contact with oxygen, light, and water. Consequently, HPβCD acts as a “protective pocket,” significantly enhancing TQ’s resistance to degradation processes. Complexation stabilizes the active compound by suppressing photodegradation (the cyclodextrin cavity restricts access to UV radiation), reducing susceptibility to oxidation, decreasing the influence of moisture, and limiting the conformational freedom of TQ, thereby stabilizing it at the energetic level. For this reason, the N2 system maintained the highest TQ percentage during accelerated stability testing.

The N3 system (PVP/PCL + TQ) shows good stability; however, its resistance is partially limited by the thermal properties of the PCL component. Polycaprolactone is more moisture-resistant and, to some extent, stabilizes TQ against photodegradation due to its semicrystalline nature and different interaction profile with TQ compared to PVP. Nevertheless, PCL has a relatively low softening temperature (approximately 58–63 °C). Under elevated temperature conditions, the PCL matrix may undergo plasticization, leading to partial fiber fusion and microstructural changes. This, in turn, may promote gradual TQ degradation and affect the structural stability of the nanofibers. Despite this, the N3 system maintains a high TQ content, particularly under moderate conditions and during UV exposure, which can be attributed to PCL’s more hydrophobic nature and its reduced moisture permeability.

**Figure 8:**
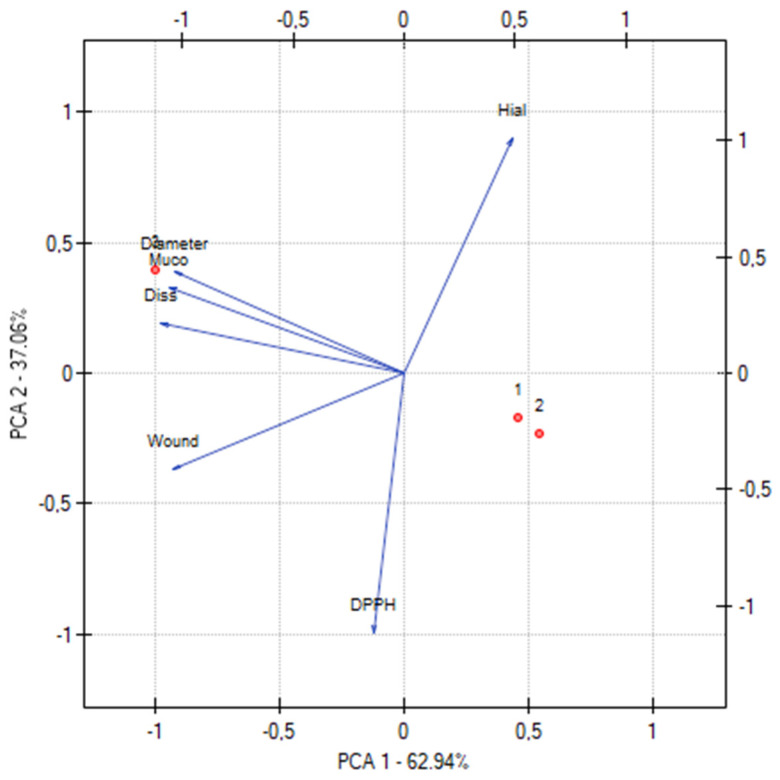
Principal component analysis (PCA) of physicochemical and biological parameters of nanofibers N1-N3, where Diameter = average nanofiber diameter (nm), Diss = time required to release 80% of TQ, Muco = mucoadhesion measured at shear rate 9.3 s^−1^, DPPH = antioxidant activity expressed as IC_50_ (mg/mL), Hial = anti-inflammatory activity (hyaluronidase inhibition), expressed as IC_50_ (mg/mL), and Wound= wound closure after 24 h (%).

Principal component analysis (PCA) suggested relationships between structural parameters, release behavior, and biological activity of the developed nanofibers (Figure 8). A very strong positive correlation was observed between fiber diameter and dissolution time (r = 0.978), indicating that thinner nanofibers required less time to release 80% of thymoquinone, whereas thicker fibers exhibited a more prolonged release profile. This is consistent with the higher surface area-to-volume ratio of thinner fibers, which facilitates faster drug diffusion. Fiber diameter was also strongly correlated with mucoadhesion (r = 0.998), and dissolution time showed a similarly strong correlation with mucoadhesion (r = 0.990). These results indicate that systems with slower drug release exhibit enhanced bioadhesive properties, likely due to the formation of a denser, more structured polymer matrix. Wound-healing efficiency showed strong positive correlations with dissolution time (r = 0.845), mucoadhesion (r = 0.761), and fiber diameter (r = 0.713), confirming that prolonged release and improved retention at the application site are key determinants of enhanced cell migration and wound closure. Biological activity parameters followed consistent trends. Antioxidant activity (DPPH IC_50_) was strongly negatively correlated with anti-inflammatory activity (hyaluronidase IC_50_) (r = −0.945), indicating that systems with lower IC_50_ values (higher activity) in the DPPH assay also exhibit stronger hyaluronidase inhibition. This confirms that antioxidant and anti-inflammatory effects are closely related. In contrast, DPPH activity showed weak or negative correlations with structural parameters, including diameter (r = −0.278), dissolution (r = −0.071), and mucoadhesion (r = −0.210), suggesting that antioxidant activity is primarily governed by thymoquinone availability rather than matrix structure. Hyaluronidase inhibition was negatively correlated with wound healing (r = −0.736), indicating that systems with stronger immediate anti-inflammatory activity are not necessarily the most effective at promoting wound closure; rather, wound closure depends more on sustained drug release and bioadhesion.

PCA distinguishes two dominant domains: (1) a structural–release domain (diameter, dissolution, mucoadhesion), and (2) a biological activity domain (DPPH and hyaluronidase IC_50_). These findings demonstrate that optimal wound-healing performance results from a balance between drug availability and controlled release, rather than from a single dominant parameter.

It should be noted that the PCA was performed using data obtained from only three formulations. Therefore, the identified relationships should be considered exploratory and interpreted with caution. The PCA was primarily used to visualize trends and potential associations between formulation characteristics and biological performance rather than to establish definitive correlations. Further studies involving a larger number of formulations are required to validate these observations and strengthen the statistical significance of the multivariate analysis.

A limitation of the present study is the absence of blank nanofiber formulations without thymoquinone. Consequently, the individual contributions of the polymer matrices to the observed biological effects, including fibroblast migration, antioxidant activity, and anti-inflammatory activity, could not be distinguished from those associated with the active compound. Future studies should include appropriate blank carrier controls to better elucidate the respective roles of the polymer matrix and thymoquinone in determining the overall biological response.

## 4. Conclusions

In this study, three electrospun nanofiber systems containing thymoquinone (TQ) and based on different polymeric matrices (PVP, PVP/HPβCD, and PVP/PCL) were successfully developed and comprehensively characterized with respect to physicochemical properties, release behavior, biological activity, and stability. All formulations formed uniform nanofibrous structures with TQ molecularly dispersed within the polymer matrix. The composition of the system significantly impacted fiber morphology, drug release kinetics, and functional performance. The incorporation of HPβCD (N2) resulted in thinner, more homogeneous fibers and significantly enhanced TQ solubility, leading to the fastest release and improved antioxidant and anti-inflammatory activity. In contrast, the presence of PCL (N3) produced thicker fibers with a more hydrophobic and structured matrix, resulting in sustained release, superior mucoadhesion, and the most pronounced wound-healing effect. Biological studies confirmed that all TQ-loaded nanofibers promote fibroblast migration and wound closure, with effectiveness increasing in the order N1 < N2 < N3. While N2 demonstrated the highest immediate biological activity due to enhanced TQ availability, N3 provided more favorable conditions for prolonged stimulation of cell migration, highlighting the importance of controlled release systems in wound-healing applications. Stability studies revealed that incorporating HPβCD significantly improved TQ’s resistance to thermal, humidity, and photolytic degradation, whereas PVP-based systems without stabilizing agents showed the lowest stability.

Principal component analysis (PCA) confirmed that the performance of the developed systems is governed by two main factors: (1) drug availability, associated with solubility and rapid release, and (2) sustained release combined with bioadhesion. Importantly, wound-healing efficiency correlated more strongly with the latter, indicating that prolonged drug presence at the application site is a key determinant of therapeutic effectiveness.

The results demonstrate that the rational selection of polymer composition enables modulation of TQ delivery profiles and biological responses. Among the tested systems, the PVP/PCL-based nanofibers (N3) appear to be the most promising candidates for wound-dressing applications, combining sustained release, high bioadhesion, and superior wound-healing performance, while the PVP/HPβCD system (N2) offers advantages in solubility and stability. These findings provide valuable insight into the design of advanced nanofibrous wound dressings for the effective delivery of poorly soluble natural compounds such as thymoquinone.

## Figures and Tables

**Table 1:** Composition of systems.

	N1 (TQ/PVP)	N2 (TQ/PVP/HPβCD)	N3 (TQ/PVP/PCL)
Solvent	10.0 mL methanol	10.0 mL methanol	10.0 mL methanol: dichloromethane (50:50 *v*/*v*)
Thymoquinone (TQ)	1.0 g	1.0 g	1.0 g
Polyvinylpyrrolidone (PVP)	2.0 g	2.0 g	1.5 g
Hydroxypropyl-β-cyclodextrin (HPβCD)	-	2.0 g	-
Polycaprolactone (PCL)	-	-	0.5 g

**Table 2:** Kinetic parameters of TQ release from nanofibers N1–N3.

	Zero-Order Kinetics		First-Order Kinetics		Higuchi Kinetics		Korsmeyer–Peppas Kinetics	
	K	R^2^	K	R^2^	K	R^2^	n	R^2^
N1	2.71	0.46	0.08	0.19	12.99	0.89	0.56	0.66
N2	7.86	0.41	0.29	0.23	18.14	0.84	0.83	0.68
N3	1.24	0.84	0.04	0.48	9.60	0.92	0.48	0.94

**Table 3:** Biological activity of nanofibers.

	Antioxidant Activity Inhibition of the DPPH Radical Activity	Anti-inflammatory Activity Inhibition of the Activity of the Enzyme Hyaluronidase
TQ	IC_50_ = 0.112 ± 0.016 mg/mL ^a^	IC_50_ = 0.203 ± 0.030 mg/mL ^a^
N1 (TQ/PVP)	IC_50_ = 0.304 ± 0.015 mg/mL ^b^	IC_50_ = 0.456 ± 0.060 mg/mL ^c^
N2 (TQ/PVP/HPβCD)	IC_50_ = 0.483 ± 0.021 mg/mL ^d^	IC_50_ = 0.305 ± 0.040 mg/mL ^b^
N3 (TQ/PVP/PCL)	IC_50_ = 0.374 ± 0.018 mg/mL ^c^	IC_50_ = 0.353 ± 0.050 mg/mL ^b^
Ascorbic acid	IC_50_ = 0.066 ± 0.013 mg/mL	-
β-escin	-	IC_50_ = 0.74 ± 0.01 mg/mL

Mean values in a column marked with the same letter are not statistically significantly different at *p* = 0.05, according to Duncan’s test.

**Table 4:** Stability assessment of TQ-loaded nanofibers N1–N3 under stress conditions.

	Test Conditions	Organoleptic Observation	TQ Content
N1 (TQ/PVP)	Increased temperature (60 °C, 0% RH)	Slight yellowing, fibers preserved	89.23 ± 2.35%
	Increased relative humidity (25 °C, 75% RH)	Minor fiber adhesion, surface tackier	77.82 ± 1.28%
	UV/Vis light exposure	Pronounced discoloration of TQ (yellowing)	65.58 ± 2.74%
N2 (TQ/PVP/HPβCD)	Increased temperature (60 °C, 0% RH)	No significant changes, fibers remained stable	96.26 ± 1.29%
	Increased relative humidity (25 °C, 75% RH)	Structure preserved, no fiber adhesion observed	94.94 ± 2.37%
	UV/Vis light exposure	No discoloration	93.13 ± 0.92%
N3 (TQ/PVP/PCL)	Increased temperature (60 °C, 0% RH)	Partial fiber thickening observed	88.93 ± 1.28%
	Increased relative humidity (25 °C, 75% RH)	Good stability with slight fiber cohesion in some areas	90.36 ± 2.19%
	UV/Vis light exposure	Moderate color changes	89.63 ± 1.93%

## Data Availability

The original contributions presented in this study are included in the article. Further inquiries can be directed to the corresponding author.
